# Devising an Indicator to Detect Mid-Term Abortions in Dairy Cattle: A First Step Towards Syndromic Surveillance of Abortive Diseases

**DOI:** 10.1371/journal.pone.0119012

**Published:** 2015-03-06

**Authors:** Anne Bronner, Eric Morignat, Viviane Hénaux, Aurélien Madouasse, Emilie Gay, Didier Calavas

**Affiliations:** 1 ANSES-Lyon, Unité Epidémiologie, 31 avenue Tony Garnier, 69364 Lyon Cedex 07, France; 2 INRA, UMR1300 Biologie, Epidémiologie et Analyse de Risque en santé animale, CS 40706, 44307 Nantes, France; 3 LUNAM Université, Oniris, Ecole nationale vétérinaire, agroalimentaire et de l’alimentation Nantes Atlantique, UMR BioEpAR, 44307 Nantes, France

## Abstract

Bovine abortion surveillance is essential for human and animal health because it plays an important role in the early warning of several diseases. Due to the limited sensitivity of traditional surveillance systems, there is a growing interest for the development of syndromic surveillance. Our objective was to assess whether, routinely collected, artificial insemination (AI) data could be used, as part of a syndromic surveillance system, to devise an indicator of mid-term abortions in dairy cattle herds in France. A mid-term abortion incidence rate (MAIR) was computed as the ratio of the number of mid-term abortions to the number of female-weeks at risk. A mid-term abortion was defined as a return-to-service (i.e. a new AI) taking place 90 to 180 days after the previous AI. Weekly variations in the MAIR in heifers and parous cows were modeled with a time-dependent Poisson model at the *département* level (French administrative division) during the period of 2004 to 2010. The usefulness of monitoring this indicator to detect a disease-related increase in mid-term abortions was evaluated using data from the 2007–2008 episode of bluetongue serotype 8 (BT8) in France. An increase in the MAIR was identified in heifers and parous cows in 47% (n = 24) and 71% (n = 39) of the *départements*. On average, the weekly MAIR among heifers increased by 3.8% (min-max: 0.02–57.9%) when the mean number of BT8 cases that occurred in the previous 8 to 13 weeks increased by one. The weekly MAIR among parous cows increased by 1.4% (0.01–8.5%) when the mean number of BT8 cases occurring in the previous 6 to 12 weeks increased by one. These results underline the potential of the MAIR to identify an increase in mid-term abortions and suggest that it is a good candidate for the implementation of a syndromic surveillance system for bovine abortions.

## Introduction

Over the past years, several major abortive disease outbreaks and epizootics have occurred in ruminant livestock in Europe. Notable examples include zoonotic diseases, such as Q fever in the Netherlands between 2007 and 2010 [1], and bovine brucellosis in Belgium and France in 2012 [2,3]. In addition to the concern for public health, epizootics of abortive diseases may also cause severe economic losses. For example, in August 2006, bluetongue serotype 8 (BT8) was detected for the first time in the Netherlands with indirect costs to the Dutch farming industry estimated at about 50 million euros per year [4]. Moreover, there is a risk that Rift Valley Fever, a major zoonotic viral disease that currently circulates in most African countries and the Arabian Peninsula [5], will spread to the southern Mediterranean Basin. In this context, bovine abortion surveillance is critical for human and animal health because it plays an important role in the early warning for several diseases.

In France, bovine abortion surveillance consists in mandatory notification. It aims to detect as early as possible any resurgence of bovine brucellosis, of which the country has been declared officially free since 2005 [6]. According to European regulations, in cases of bovine abortion, farmers must consult their veterinarian who reports it to State veterinary services and samples the aborted cow for serological analysis of *Brucella* [7,8]. Abortion is defined by national regulations as the expulsion of the fetus or calf, either stillborn or which dies less than 48 hours after being born [9]. However, only some abortions are notified. First, reported abortions are mainly late abortions because early abortions are usually considered as fertility problems and mid-term abortions, which occur between the third and sixth month of pregnancy, are rarely detected by farmers. Moreover, in a previous study using capture-recapture methods, we estimated that the overall sensitivity of the system, i.e. the proportion of farmers who reported abortion(s) among farmers who were assumed to have detected abortion(s), was about 20% for beef and 39% for dairy cattle herds [10].

Lack of sensitivity is often cited as one of the main limitations of event-driven surveillance systems (i.e. “clinical” or “passive” surveillance systems) [11], and raises concern about the ability of such systems to effectively provide early detection of health events. In this context, there is a need to improve traditional surveillance systems, but also to explore other ways to improve animal and public health surveillance. One of these ways is syndromic surveillance [12], defined as “the real-time (or near real-time) collection, analysis, interpretation, and dissemination of health-related data to enable the early identification of the impact (or absence of impact) of potential human or veterinary public-health threats which require effective public health action” [13]. Syndromic surveillance is not based on laboratory-confirmed diagnoses but on non-specific clinical signs, symptoms and proxy measures (or “syndromes”) [13]. In regard to abortive diseases, we evaluated the possibility of using artificial insemination (AI) data to develop an indicator for syndromic surveillance. These data, routinely collected for purposes other than surveillance, have already been used to analyze the influence of several diseases on reproductive disorders [14–17]. In particular, an analysis of the time elapsed between successive AIs confirmed the influence of BT8 exposure during gestation on return-to-service [16,17], previously suggested by field observations [18,19] and supported by the transplacental transmission capacity of BT8 [20,21]. More recently, a study highlighted that reproduction data can be used prospectively as indicators of disease emergences [22].

In this context, our objective was to assess whether AI data could be used, as part of a syndromic surveillance system, to devise an indicator of mid-term abortions in dairy cattle herds in France in order to assess retrospectively the impact of an abortive disease. Based on the availability of data on the BT8 epizootic that emerged in France in 2006 and then spread across the country, we assessed the capacity of the indicator to identify a BT8-related increase in abortion rates, and quantify the influence of the BT8 epizootic on this indicator. We discuss the potential of the monitoring of this syndromic indicator as a complementary system for the surveillance of abortive diseases.

## Materials and Methods

### Data

Demographic and reproduction data were extracted from the French National Genetic Information System (SNIG). SNIG is a database managed by the French National Institute for Agricultural Research (INRA—*Institut national de la recherche agronomique*), in collaboration with the French Livestock Institute (*Institut de l’Elevage*). The compiled data include animal identifications and pedigrees, performance testing, insemination centers, herd books, laboratories, slaughterhouses, etc. These data were provided by INRA and obtained from herds registered for monitoring milk production volumes and in which artificial insemination (AI) is practiced; these herds represent about 60% of French dairy cattle herds. For each female, data included farm location (i.e. *département*, an intermediate French administrative division with a mean area of 5,800 km^2^), individual characteristics (identification number, birth date), reproductive events (AI and calving dates), and death date if the animal had died. Females were divided into two groups according to the number of calvings: heifers (females with no calving reported before the considered AI date) and parous cows (females with at least one calving reported before the considered AI date). Data have been gathered since 2001 but cattle registered since 1 January 2004 were selected for the study, because the notification system is considered to have been fully operational since then. Six reproductive seasons between 1 August 2004 and 31 July 2010 were used to follow the seasonality of calvings which peak in September/October. Each reproductive season X/X+1 starts on 1 August of year X and ends on 31 July of year X+1: e.g. the first reproductive season 2004/2005 started on 1 August 2004 and ended on 31 July 2005 and the last reproductive season 2009/2010 started on 1 August 2009 and ended on 31 July 2010.

During the BT8 epizootic, BT8 surveillance relied on active surveillance (based on serological sampling in herds randomly selected) and event-driven surveillance (or “passive” surveillance, based on the mandatory notification of suspected cases of BT8). Notifications of BT8 clinical cases from 2007 to 2009 in France were provided by the French Ministry of Agriculture. In contrast to active surveillance, event-driven surveillance identifies (a proxy of) the date of exposure of herds. The event-driven surveillance system requires each cattle owner to report every clinically suspect case to their veterinarian, who samples the suspected animal for confirmation. Data included the farm location (*département*), and dates of clinical suspicion and of confirmation of each BT8 case.

### Indicator of mid-term abortions

Mid-term abortion in a female was defined as the occurrence of an AI 90 to 180 days after a previous AI. For each studied *département* and female parity group (heifers versus parous cows), the number of mid-term abortions on day *d* of the study period was estimated as the number of females inseminated on day *d* who had previously been inseminated 90 to 180 days prior; the number of females at risk of having a mid-term abortion was estimated as the number of females still alive on day *d* and having been inseminated 90 to 180 days prior.

**Fig 1:**
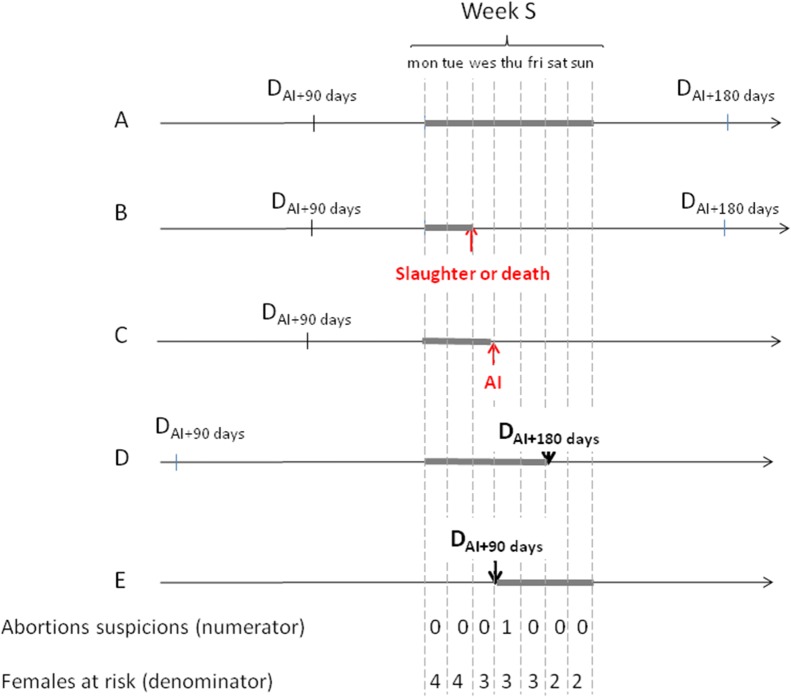
Weekly calculation of the MAIR. Suppose a situation in which five females A, B, C, D and E are recorded over week *w* at different stages of reproduction (with the same parity and in the same *département*). The period between D_AI+90 days_ and D_AI+180 days_ starts 90 days and ends 180 days after the first AI. By aggregating the number of mid-term abortions and the number of females at risk of having a mid-term abortion on a weekly timescale, one mid-term abortion was identified out of three female-weeks (i.e. 21 female-days/7) at risk.

To avoid the weekday effect in the modeling process, data were aggregated for each *département* and female parity group on a weekly timescale. Most of the females are inseminated on a week day and the number of AIs decreases sharply on Sundays and legal holidays. The number of female-weeks at risk of having a mid-term abortion in a given week was obtained by adding together the number of days of presence of each female that week divided by 7. The mid-term abortion incidence rate (MAIR) was computed on a weekly timescale as follows:

$${\text{MAIR}}_{ijw}=\frac{Y_{ijw}}{N_{ijw}}$$

where *Y*_*ijw*_ is the number of mid-term abortions over week *w* in *département i* and female parity group *j* and *N*_*ijw*_ is the number of female-weeks at risk of having a mid-term abortion. The principle behind the computation is explained in Fig. 1.

The study focused on those *départements* that reported at least 60,000 heifer-weeks and 180,000 cow-weeks at risk of having a mid-term abortion during the study period. To specifically study the effect of the BT8 epizootic, the study focused on *départements* where at least one clinical case due to BT8 had been reported in 2007 or 2008 but none due to BT1, another bluetongue serotype that emerged in the same period in the South of France [23].

### Modeling of MAIR time series

Fluctuations in MAIRs for each *département* and female parity group over the study period were modeled using a Poisson regression model with over-dispersion. All statistical analyses were computed using R [24]. The development of the regression model was carried out in two steps: the MAIR time series were first analyzed by integrating time covariates in the model and then a BT8 covariate.

#### Time covariates

For each *département* and female parity group, three models were tested successively including: (1) a linear trend with annual periodicity, (2) a linear trend with annual and six-month periodicities, and (3) a linear trend with annual, six-month and three-month periodicities. To reduce the effect of outliers on the estimate of covariate coefficients, a second round of estimations was performed, weighting the observations by the inverse of their residuals, as proposed by Farrington et al. [25]. Reweighting was conducted using the R “surveillance” package [26]. The most complete model included time covariates as follows:

$$\begin{array}{l} Log(\mu_{ijw})=\beta_{0}+\beta_{1}\times w+\beta_{2}\times \text{sin}(2\pi \times w/52)+\beta_{3}\times \text{cos}(2\pi \times w/52)+\beta_{4}\times \text{sin}(2\pi \times w\times 2/52)\\ +\beta_{5}\times \text{cos}(2\pi \times w\times 2/52)+\beta_{6}\times \text{sin}(2\pi \times w\times 4/52)+\beta_{7}\times \text{cos}(2\pi \times w\times 4/52)+\text{log}({\text{N}}_{ijw}) \end{array}$$

with *μ*_*ijw*_ representing the mean number of mid-term abortions for each week *w*, *département i* and parity group *j*, log(N_*ijw*_) as the number of female-weeks at risk of having a mid-term abortion, and β the covariate coefficients.

Model selection was conducted using the R “bblme” package where the quasi-AIC criterion (QAIC) was chosen for model parsimony [27,28]. For each *département* and female parity group, the QAICs of the three models were calculated by using the quasi-likelihood, which was the likelihood divided by the over-dispersion parameter estimated from the most complex model, i.e. the model that included a linear trend with annual, six-month and three-month periodicities [28]. The model with the lowest QAIC was selected and named M1.

#### BT8 covariate

The associations between the observed MAIR and the mean number of BT8 cases were investigated over alternative time intervals. To meet this objective, we adapted cross-correlation maps introduced by Curriero et al. [29]. This method is used to study associations between a time series of a dependent variable Y_*t*_ and a covariate X̅_*t−ℓ,t−k*_ averaged over the time interval ranging from *t* − *ℓ* to *t* − *k*, with *ℓ* ≥ *k* [29–31].

In our study, for each *département* and female parity group, we studied associations between the time series of the dependent variable MAIR_*ijw*_ and a bluetongue covariate B̅t_*w−ℓ_ij_,w−k_ij_*_ that corresponded to the number of BT8 cases averaged over the time interval ranging from week *w* - *ℓ*_*ij*_ to week *w* - *k*_*ij*_ (with *ℓ*_*ij*_ ≥ *k*_*ij*_). This bluetongue covariate was computed for all cattle herds (whatever their production type) to reflect the level of exposure of cattle herds included in the study and overcome the issue of the under-reporting of BT8 clinical cases. This covariate was included in the pre-selected model M1 as follows:

$$Log(\mu_{ijw})=\text{M}1+\beta \times \bar{\text{B}}{\text{t}}_{w-\ell_{ij},w-k_{ij}}$$

Time lags *ℓ*_*ij*_ and *k*_*ij*_ ranging from 0 to 24 weeks were tested. The BT8 virus does not cause abortions in females infected before the AI [16], and the MAIR included females up to 180 days (24 weeks) after AI. When *ℓ*_*ij*_ = *k*_*ij*_, the MAIR over week *w* depended on the number of BT8 clinical cases reported during the *ℓ*_*ij*_^th^ week (or the *k*_*ij*_^th^ week) preceding week *w*.

#### Model selection

In total, for each *département* and female parity group, 300 models *m*_*ij*_ were implemented with alternative *ℓ*_*ij*_ and *k*_*ij*_ values ranging from 0 to 24 weeks and *ℓ*_*ij*_ ≥ *k*_*ij*_. As for model M1, a second round of estimations was performed for each model *m*_*ij*_, weighting the observations by the inverse of their residuals [25]. The most parsimonious model (i.e. with the lowest QAIC) for each *département* and female parity group was named M2. Partial autocorrelations between residuals of the most parsimonious models M2 were examined in order to identify any signs of non-randomness [32]. Moreover, the fit of the M2 models was assessed graphically by verifying that the weekly observed abortion rates overlapped with the 95% prediction intervals of the MAIR, computed using the method proposed by Farrington et al. [25].

To incorporate the uncertainty of model selection into the process of time lag selection, the QAIC value of each model *m*_*ij*_ was compared to QAIC _M2_ as follows: Δ_m_ij__ = QAIC_m_ij__ −QAIC_M2_. Candidate models were defined as models with Δ_m_ij__ ≤ 2. Their relative quasi-likelihood Δ_m_ij__ was computed and normalized to unity by [33]:

$$wg_{m_{ij}}=\frac{\text{exp}(-\Delta_{m_{ij}}/2)}{\sum_{n_{m}{}_{ij}}^{}\text{exp}(-\Delta_{m_{ij}}/2)}$$

where *n_m_ij__* denotes the number of candidate models for *département i* and female parity group *j*. The resulting weights *wg_m_ij__* can be interpreted as the weight of evidence in favor of model *m*_*ij*_ being most appropriate, given both the data and the model set [34]. They were used to incorporate the uncertainty of model selection into the process of selection of time lags *ℓ*_*ij*_ and *k*_*ij*_. Time lags *ℓ_m_ij__* and *k_m_ij__* selected by each candidate model *m*_*ij*_ to compute the BT8 covariate were weighted by *wg_m_ij__* as follows:

$$\ell_{m_{ij}}^{'}=wg_{m_{ij}}\times \ell_{m_{ij}}\;\text{and}\;k_{m_{ij}}^{'}=wg_{m_{ij}}\times k_{m_{ij}}$$

Within each parity group, *départements* for which the marginal probability density functions of weighted time lags $\ell_{m_{ij}}^{'}$ and $k_{m_{ij}}^{'}$ were multimodal, with the probability value of the second best mode higher than half of the value of the major mode, were excluded.

#### Evaluation of the effect of BT8 on the MAIR

To study the influence of the mean number of BT8 cases computed over *département*-specific time intervals on the MAIR, the aggregate BT8 covariate effect for *département i* and female parity group *j* corresponded to the effect estimated by candidate models (i.e. positive: an increase in the MAIR; negative: an decrease in the MAIR; non–significant: no change in the MAIR) when all candidate models estimated the same BT8 covariate effect. When the BT8 covariate effect differed among candidate models, the aggregate BT8 covariate effect was considered as inconclusive.

To compare the influence of the number of BT8 cases on the MAIR among *départements*, the mean of weighted time lags $\ell_{m_{ij}}^{'}$ and $k_{m_{ij}}^{'}$ computed for each *département* were averaged among *départements*, distinguishing between heifers and parous cows, resulting in average time lags *ℓ̅_j_* and *k̅_j_*. Then, for each *département*, the effect of the mean number of BT8 cases between *w* − *ℓ̅_j_* and *w* − *k̅_j_* on the MAIR was predicted from a model (named M3) that included the BT8 covariate B̅t_*w−ℓ̅_j_;w−k̅_j_*_ for the corresponding female parity group. The relative risk of bluetongue on the MAIR was then estimated for *département i* and female parity group *j* as *RR*_*ij*_ = exp(*β*_*ij*_) [35].

## Results

### Population characteristics

**Fig 2:**
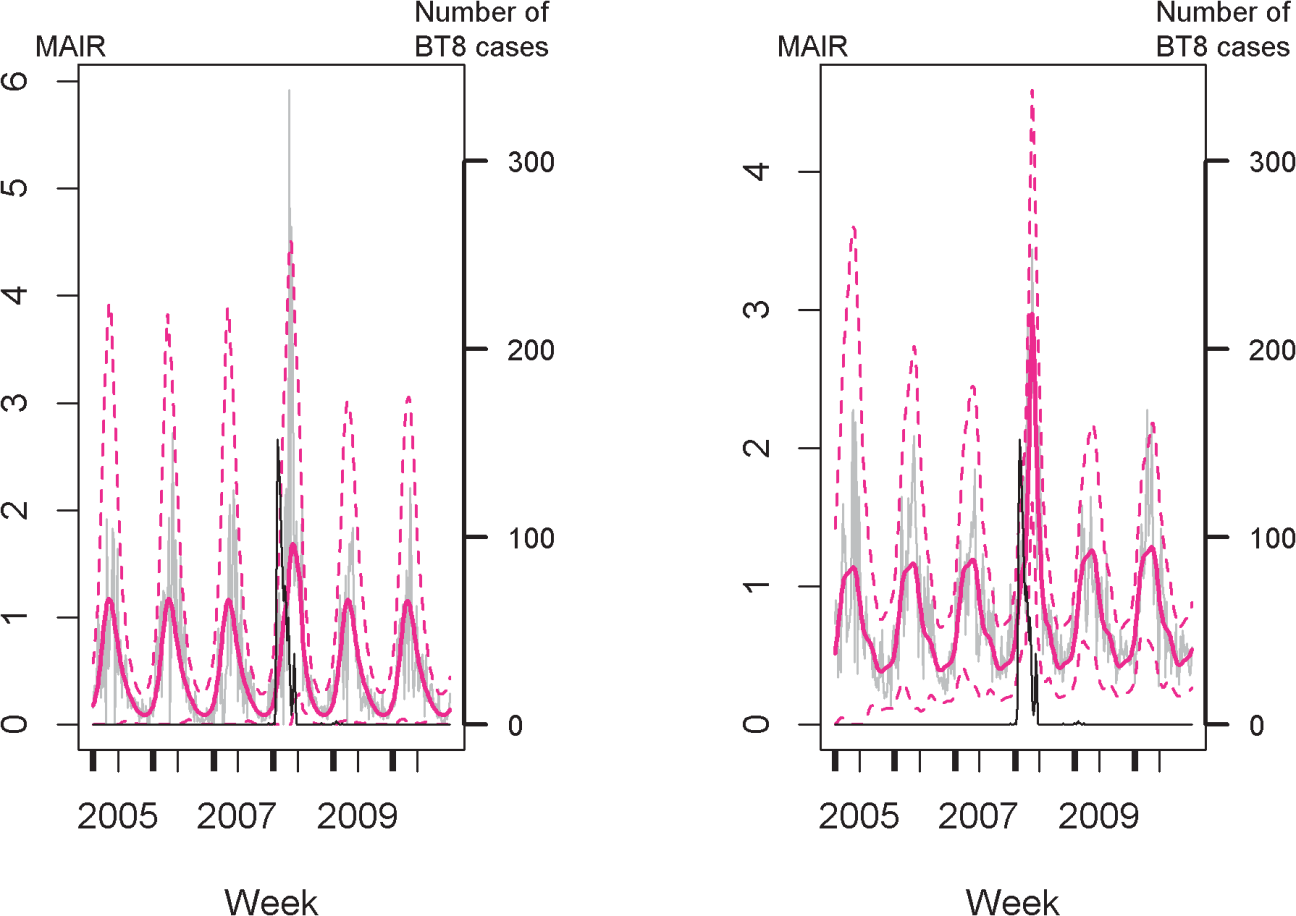
Observed and predicted weekly MAIR and number of BT8 cases in the Aisne *département*. The observed mid-term abortion incidence rate, MAIR_*ijw*_ (in grey), was computed for heifers (Fig. 2A, on the left) and parous cows (Fig. 2B, on the right) as the ratio of the observed number of mid-term abortions to the number of at-risk female-weeks. The predicted MAIR (solid pink line) and predictive intervals (dashed pink line) were estimated by modeling the MAIR according to time and the BT8 covariate averaged over a *département*-specific time interval (with the candidate model having the lowest QAIC, M2). The number of clinical BT8 cases is plotted in black.

The study population included 147,517,197 female-weeks from 59 *départements* that met the inclusion criteria (varying from 268,906 to 12,629,289 female-weeks among *départements*), 30% of which were heifers. During the study period, 847,991 mid-term abortions were estimated: 176,306 among heifers and 671,685 among parous cows. Among heifers, the observed MAIR averaged over the study period was less than 0.32 per 100 female-weeks in 25% of the *départements*, less than 0.38 per 100 female-weeks in 50% of the *départements*, and less than 0.44 per 100 female-weeks in 75% of the *départements* (with a mean value of 0.38 per 100 female-weeks). Among parous cows, the observed MAIR averaged over the study period was less than 0.55 per 100 female-weeks in 25% of the *départements*, less than 0.67 per 100 female-weeks in 50% of the *départements*, and less than 0.75 per 100 female-weeks in 75% of the *départements*. For each *département* and female parity group, the observed MAIR varied periodically with higher values in November and December (Fig. 2).

In total, 29,696 BT8 clinical cases were notified in the 59 *départements* during the study period. The first epizootic wave spread across 36 out of the 59 selected *départements*, peaking in September 2007 (with 3,778 outbreaks). All 59 *départements* were infected during the second epizootic wave, peaking in August 2008 with 7,596 outbreaks.

### Selection of candidate models

The pre-selected model M1 included a linear trend with an annual periodicity in 16 *départements* for heifers and 9 *départements* for parous cows; a linear trend with annual and six-month periodicities in 18 *départements* for heifers and 11 *départements* for parous cows; a linear trend with annual, six-month and three-month periodicities in 25 *départements* for heifers and 39 *départements* for parous cows.

Among heifers, between 1 and 300 models provided a good fit to the data (Δ_m_ij__ ≤ 2), 45 *départements* having less than 50 candidate models. Among parous cows, 1 to 174 models were selected per *département*, 48 *départements* having less than 50 candidate models. Residuals from the most parsimonious M2 models were not auto-correlated. Comparison of the weekly observed and predicted MAIR confirmed their good fit (Fig. 2); however, the observed MAIR exceeded the 95% prediction intervals over a few weeks (between 1 and 13 out of the 312 weeks included in the study period, depending on *départements*). Eight *départements* were excluded for heifers and four *départements* for parous cows because the marginal probability density functions of $\ell_{m_{ij}}^{'}$ and $k_{m_{ij}}^{'}$ were multimodal, and the probability value of the second-best mode was higher than half of the value of the major mode.

### Influence of the BT8 covariate on the MAIR

**Fig 3:**
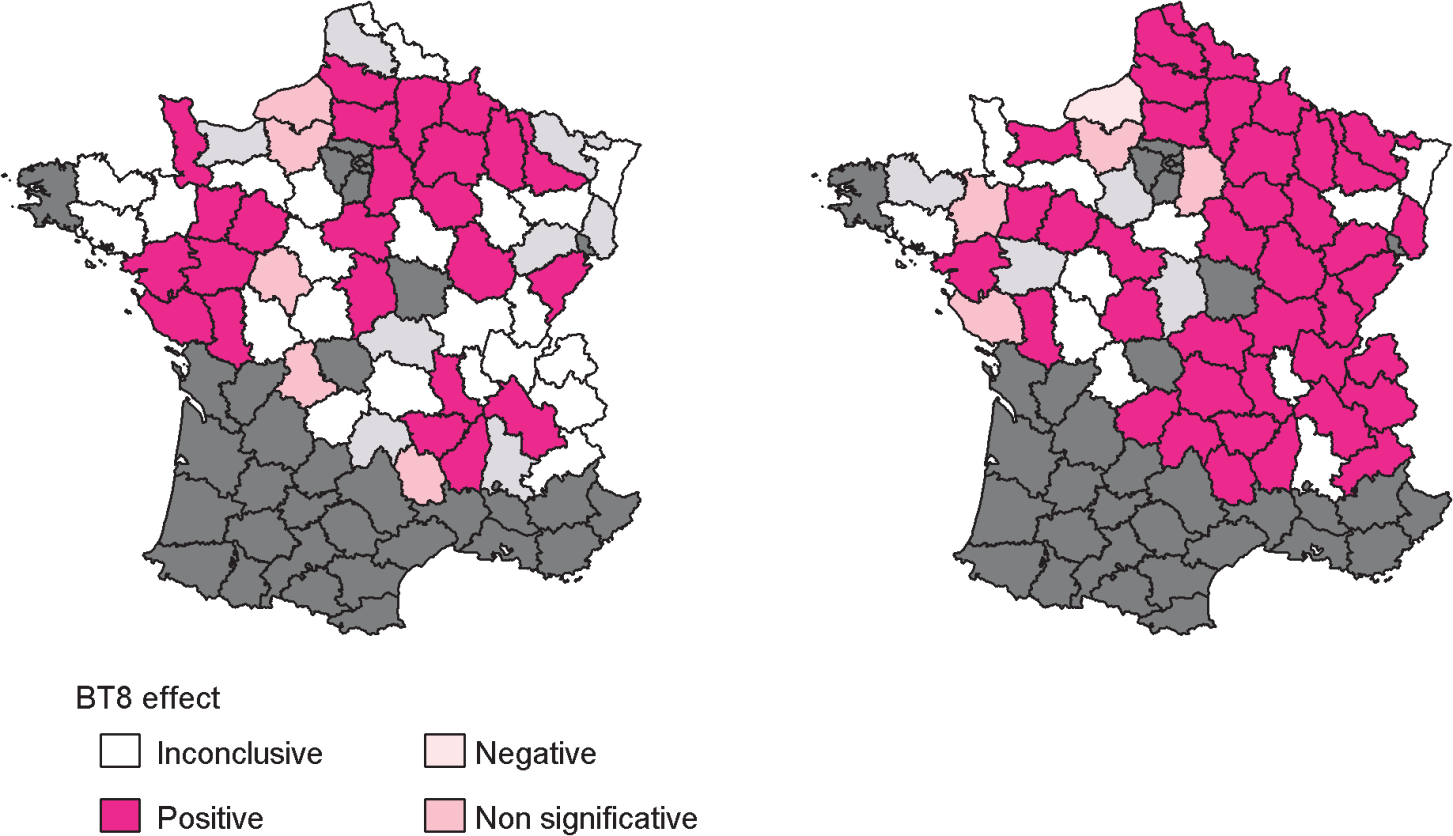
Effect of BT8 on the MAIR by *département*. *Département*-level effects are shown for heifers (Fig. 3A, on the left) and parous cows (Fig. 3B, on the right). *Départements* in dark gray were excluded because they reported BT8 and BT1 cases; *départements* in light gray are those excluded from the study because the marginal probability density functions of $\ell_{m_{ij}}^{'}$ and $k_{m_{ij}}^{'}$ were multimodal, with the probability value of the second best mode higher than half of the value of the major mode. The effect of the mean number of BT8 cases during the *département*-specific weighted average time interval on the weekly MAIR was positive in *départements* in dark pink, non-significant in *départements* in light pink, and negative in *départements* in pale pink.

**Fig 4:**
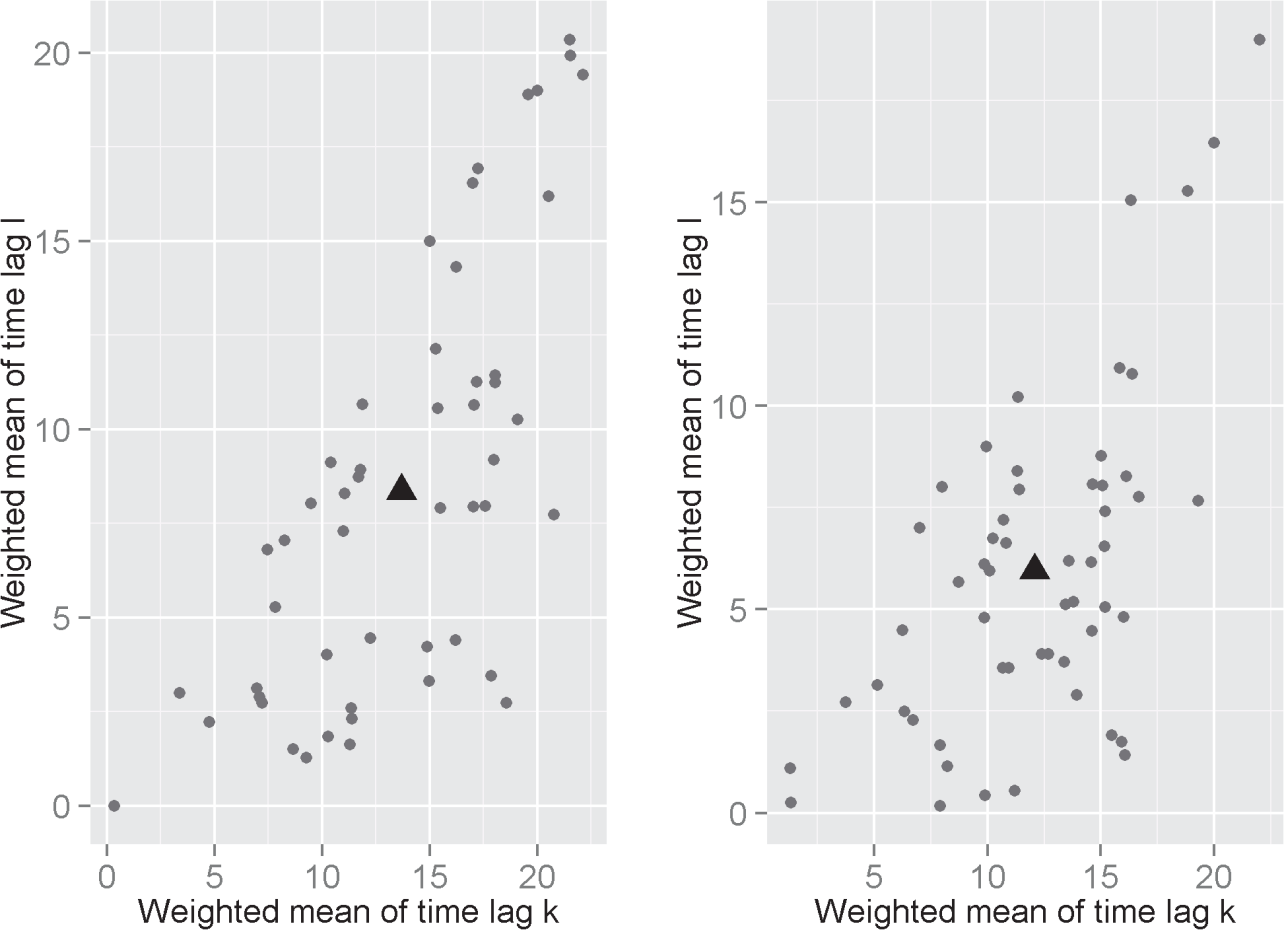
Distribution of *départements* according to their weighted mean of time lags *ℓ_m_ij__* and *k*_m_ij__. Each graph represents the distribution of the 51 *départements* (among heifers, Fig. 4A, on the left) and 55 *départements* (among parous cows, Fig. 4B, on the right) depending on their weighted mean of time lags *ℓ_m_ij__* and *k*_m_ij__. Time lags *ℓ̅_j_* and *k̅_j_* averaged among *départements* are also plotted (black triangle).

The effect of the mean number of BT8 cases computed over *département*-specific time intervals showed an increase in the MAIR in 47% out of the 51 *départements* among heifers and 71% out of the 55 *départements* among parous cows (Table 1 and Fig. 3). It showed an increase in the MAIR in 18 *départements* among both groups of females, as illustrated by the Aisne *département* (Fig. 2A and 2B). Among heifers, the mean of weighted time lags ranged from 0 to 22 weeks for $\ell_{m_{ij}}^{'}$ and from 0 to 20 weeks for $k_{m_{ij}}^{'}$ among *départements* (Fig. 4); weighted mean of time intervals varied from 0 to 15 weeks. Among parous cows, the mean of weighted time lags ranged from 0 to 22 weeks for $\ell_{m_{ij}}^{'}$ and for 0 to 19 weeks for $k_{m_{ij}}^{'}$ among *départements* (Fig. 4); weighted mean of time intervals varied from 0 to 14 weeks.

**Table 1:** Qualitative effect of the BT8 covariate on the MAIR computed over *département*-specific and common time intervals.

		BT8 covariate effect computed over a common time interval					
		Heifers			Parous cows		
		Positive	Non-significant	Total	Positive	Non-significant	Total
BT8 covariate effect computed over *département*-specific time intervals	Positive	19	5	24 (47%)	36	3	39 (71%)
	Non-significant	1	4	5 (10%)	0	4	4 (7%)
	Negative	0	0	0%	0	1	1 (2%)
	Inconclusive	11	11	22 (43%)	0	11	11 (20%)
	Total	31 (61%)	20 (39%)	51 (100%)	36 (65%)	19 (35%)	55 (100%)

This contingency table illustrates, for each female parity group, the distribution of results on the bluetongue effect on the MAIR among *départements* based on *département*-specific time intervals (from *w* − *ℓ_m_ij__* to *w* − *k_m_ij__* before week *w*) versus the common time interval (from *w* − *ℓ̅_j_* to *w* − *k̅_j_*). The common time interval ranged from 8 to 13 weeks before week *w* for heifers and from 6 to 12 weeks for parous cows. See Methods for details.

**Fig 5:**
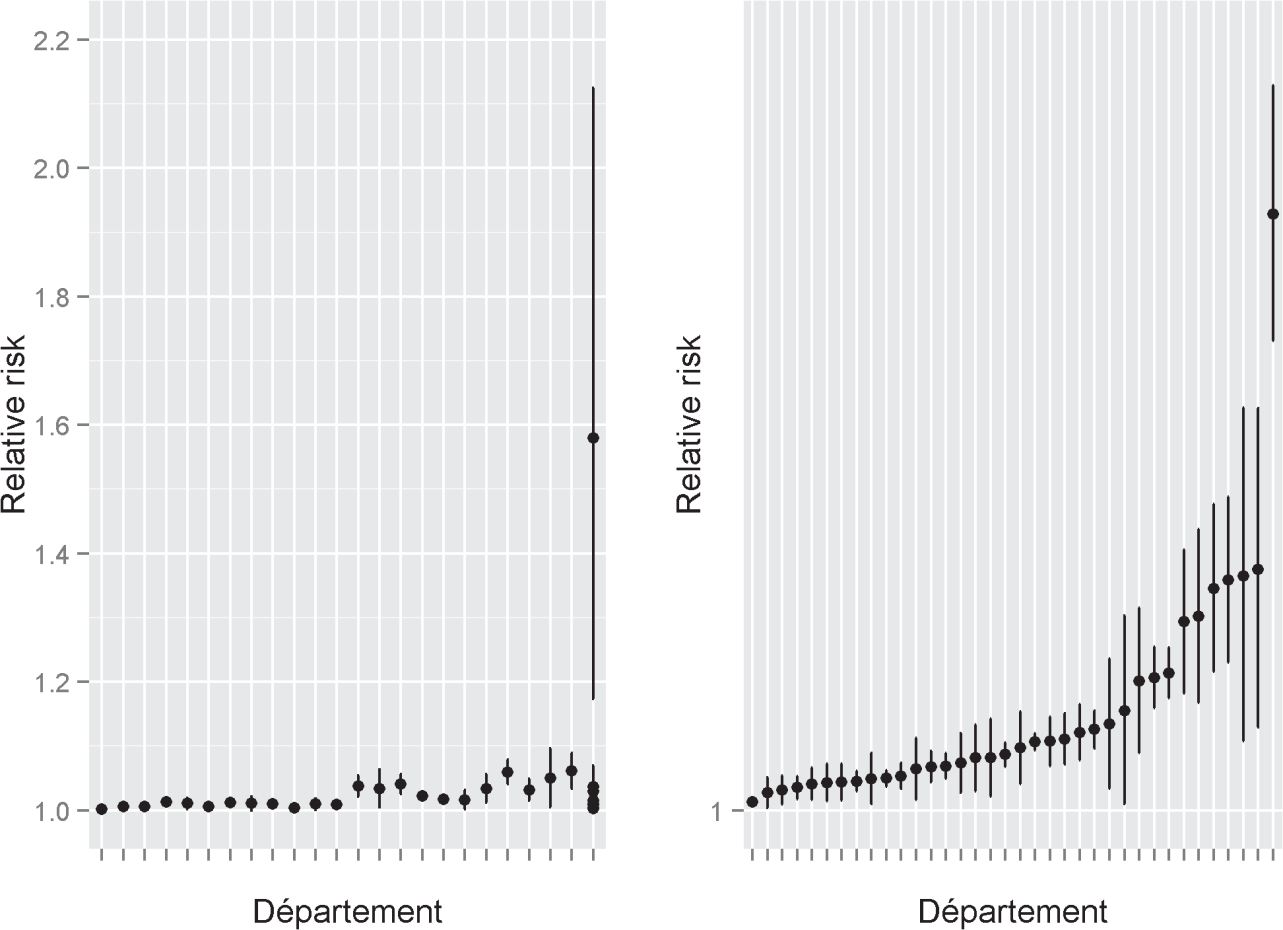
Relative risk and confidence interval of the bluetongue covariate computed over common time intervals. Relative risks (RR) and confidence intervals of the bluetongue covariate are displayed for the *départements* in which the bluetongue covariate had a positive effect, for heifers (Fig. 5A, on the left, 31 *départements*), and for parous cows (Fig. 5B, on the right, 36 *départements*). Départements are sorted in the ascending order of their RR. In the other *départements*, the bluetongue covariate had a non-significant effect.

Among heifers, time lags *ℓ̅_j_* and *k̅_j_* averaged among *départements* were of 13 and 8 weeks (Fig. 4). The number of BT8 cases averaged over a common time interval ranging between 8 and 13 weeks prior to week *w* (B̅t_*w−13,w−8*_) had a positive effect in 61% of *départements* (Table 1), with a relative risk (RR) ranging from 1.002 to 1.579 and a median and mean of 1.013 and 1.038, respectively (Fig. 5). Among parous cows, average time lags *ℓ̅_j_* and *k̅_j_* estimated among *départements* were of 12 and 6 weeks (Fig. 4). The number of BT8 cases averaged over a common time interval ranging between 6 and 12 weeks prior to week *w* (B̅t_*w−12,w−6*_) had a positive effect in 65% of *départements* (Table 1), with an RR ranging from 1.001 to 1.085 and a median and mean value of 1.008 and 1.014, respectively (Fig. 5).

## Discussion

Using AI data, we studied the incidence rate of mid-term abortions, a potential indicator for developing and implementing a syndromic surveillance system of abortive diseases in cattle. We modeled temporal variations of the MAIR in heifers and parous cows during 2004–2010 in the 59 *départements* that were impacted by the BT8 epizootic in 2007–2008. Our retrospective approach highlighted that the mean number of BT8 cases over a *département*-specific time interval was related to an increase in the weekly MAIR in 47% of the *départements* for heifers and 71% of the *départements* for parous cows. Overall, we found that the MAIR in heifers was most likely influenced by the mean number of BT8 cases reported 13 to 8 weeks prior, with a mean increase of 3.8% of the MAIR (min-max: 0.02–57.9%). In parous cows, the MAIR increased by 1.4% on average (0.01–8.5%) when the number of BT8 cases averaged over the preceding 12 to 6 weeks increased by one.

### Limitations of using AI data for syndromic surveillance

The MAIR can be used as a proxy of the actual rate of mid-term abortion occurrence in the population. Nonetheless, several types of abortion events are not captured in the MAIR. For example, based on the analysis of the mandatory abortion notification data, we estimated that 25% to 40% of cows who abort between the sixth and the ninth month of pregnancy are slaughtered without any re-insemination (data not shown). In addition, the MAIR does not include mid-term abortions that are followed by an extended delay before re-insemination. On the other hand, the return-to-service period between 90 to 180 days may include some embryonic mortalities or early abortions, because re-insemination occurs at least 21 days after the abortion (minimum time for the cow to return to estrus). Time lapses between a reproductive disorder and re-insemination also depend on herd management. The increase in the MAIR in November-December, i.e. about two months after the peak of the calving season, reflects the willingness of farmers to re-inseminate aborting cows at the same time as other cows.

As a candidate indicator for syndromic surveillance, the MAIR should be sensitive and specific to changes in the number of mid-term abortions in the population [36,37]. In particular, we made the assumption that seasonal variation due to events other than mid-term abortions (such as the probability for a cow to be re-inseminated or culled) is constant over years. However, during the second semester of 2007, the probability of a dairy cow being culled (possibly including females inseminated over the 90 to 180 previous days) likely decreased with an increase in milk quotas (the amount that farmers are allowed to produce) [38]. This seasonal variation may have slightly altered the sensitivity of the MAIR if the decrease in the probability of being culled differed between aborting cows (especially dry cows) and non-aborting cows. The weak influence of this seasonal variation on the MAIR was partially confirmed by the absence of residual auto-correlation and by the good fit of the models.

Our objective was to assess whether MAIR could identify the introduction or spread of an abortive event with a significant economic impact, i.e. that affects a large number of cattle herds. We used the BT8 epizootic in France in 2007 and 2008 to assess the ability of the MAIR to identify an increase in abortions. Some variations in the MAIR may have been due to other abortive diseases (such as neosporosis, salmonellosis that are enzootic in France) and thus, remained unexplained by our model. Nonetheless, because BT8 clinical cases are under-reported [39,40], the influence of BT8 on the MAIR was likely under-estimated.

### Ability of the MAIR to reflect BT8-related increases in abortion

By using a multiplicative Poisson model, we assumed that the relationship between the number of mid-term abortions and the number of cattle herds clinically infected by BT8 was exponential. Indeed, the prevalence of infected animals estimated in *département i* during a week *w* equaled the prevalence of infected herds multiplied by the within-herd prevalence. Based on a previous study, within-herd seroprevalence rate increased as herd-level seroprevalence rate increased during the BT8 epizootic [39]. Indeed, the disease certainly continued spreading within infected herds after their identification. In our study, the number of mid-term abortions was likely to be proportional to the number of infected animals, and the number of cattle herds clinically infected by BT8 to be proportional to the total number of infected cattle herds. Therefore, as BT8 spread across *département i*, the number of clinical herds increased, and the number of mid-term abortions increased exponentially (as within-herd prevalence also increased).

Our results indicated that BT8 cases had a delayed effect of about 13 to 8 weeks in heifers and 12 to 6 weeks in parous cows. This increase in the MAIR of at least six weeks (i.e. 42 days) for parous cows and eight weeks (i.e. 56 days) for heifers after the occurrence of BT8 cases concerns females inseminated up to 180 days prior to week *w*. Thus, the risk of mid-term abortion increased for females infected by BT8 during the 19–20 weeks following insemination in heifers or during the 17–18 weeks following AI in parous cows. This level of risk is consistent with a previous case-control study that concluded that BT8 exposure during the first three months of gestation was associated with a higher increase in return-to-service 90 to 200 days after an initial AI compared to BT8 exposure after the third month of gestation [16].

On average, we found that the MAIR computed for heifers increased by 3.8% when the average number of BT8 cases in the preceding 8 to 13 weeks increased by one, and by 1.4% in parous cows. These effects did not differ much from those estimated by Nusinovici et al. [16], who estimated that the mean effect in BT8-exposed areas corresponded to an increase of 1.16 to 1.78% of return-to-service 90 to 200 days after a previous AI for cows with no return within 90 days, compared to cows in non-infected herds.

Our results showed that the BT8 effect varied among *départements*, in terms of the amount of time elapsed (when the BT8 covariate was computed over *département*-specific time intervals, Fig. 4) and the force of the effect (when the BT8 covariate was computed over a common time interval). These variations may be due to differences in under-reporting of BT8 clinical cases among *départements*. Even if farmers were required to report every clinically suspect case to their veterinarian, we supposed that farmers’ awareness about disease risk differs among *départements*. Moreover, some farmers are reluctant to report BT8 clinical suspicions because they fear social and economic consequences [41]. In addition, the probability of infected herds being reported was certainly higher in 2008 than at the onset of the epizootic in 2007, due to the implementation of a financial compensation plan for deceased animals in late 2007 that was increased in 2008. On the other hand, the amount of time elapsed between a first AI and re-insemination after a mid-term abortion depends on the timing between the first AI and BT8 exposure, which varied among *départements*. Thus, the effect of BT8 cases is likely to be over-estimated in *départements* with high under-reporting of BT8 cases, and further delayed in *départements* with a long period between AI and BT8 exposure, or between exposure and re-insemination.

### Heterogeneity of the BT8 effect on MAIR among départements

Our study showed that, in some *départements* (eight in heifers and four in parous cows), long time lags provided a similar fit to the data (models with ΔAIC ≤ 2), and in some cases, the results related to the effect of the number of BT8 cases on the MAIR diverged (i.e. from positive to non-significant or negative). It is possible that for some time lags the effect on MAIR was not due to the bluetongue epizootic but to other factors (such as the occurrence of other diseases or a modification in herd management). In addition, given the high number of time lags tested (n = 300), type I errors were expected and may explain the divergence observed in results among models for these *départements*. However, results from alternative suitable models were convergent in the majority of *départements* and the flexibility of our approach regarding the selection of the value for the BT8 covariate made it possible to select the most appropriate time lag for each *département*.

Second, we found that the BT8 effect was not significant or was negative in a few *départements*. *Départements* where no effect was predicted had not reported a high number of BT8 cases (less than 837 BT8 cases), suggesting a lack of statistical power. The negative effect is difficult to explain but might be due to modifications in herd management during the BT8 epizootic that caused an increase in the lag between reproductive disorders and re-insemination (exceeding 180 days).

Third, the common time lag for all *départements*, computed as the mean of the *département*-selected time lags, may not be the most appropriate in some *départements*; however, the QAIC difference between models M3 and M2 was less than 10 in 90% of the *départements*.

### Implications of our findings for syndromic surveillance

Previous studies based on the Cox model have suggested that AI data could be used to evidence an increase in reproductive disorders or under-reporting during an epidemic [16,22,40]. However, to our knowledge, our study is the first to suggest that AI data could be used for implementation of a syndromic surveillance system retrospectively. This type of system can contribute to the assessment or early identification of a health impact or absence of an impact of potentially health-threatening incidents [42]. There are however some caveats.

First, as previously underlined, the MAIR is not necessarily related to a specific abortive event and can identify any reproductive disorder. Abortions can be due to exotic (such as brucellosis, Rift Valley Fever) or enzootic (such as neosporosis, salmonellosis, [43]) diseases, or to intoxications or metabolic disorders [44–47]. Any increase in MAIR must be investigated in the field to confirm the actual increase in mid-term abortions and identify the cause.

Second, our indicator cannot cover the entire bovine population at risk in France because it would focus only on calvings registered with the SNIG, representing about 50% of calvings and 60% of French dairy cattle herds. Last, although AI data are automatically and routinely collected in real time, the MAIR detects mid-term abortions and thus, any introduction of an exotic abortive disease with a time lag of at least three weeks (necessary time for the cow to return to estrus). Timeliness depends on the time between exposure to the abortive agent and re-insemination of the aborted cow, which is a combination of several time lags. The incubation period (i.e. between exposure and abortion occurrence) is disease-specific and also depends on the gestation stage [16]. Thus, this lag varies from 2 to 18 days after BT8 exposure [48] and is highly variable in cases of brucellosis infection [49]. The lag between abortion occurrence and detection depends on farmers’ practices, herd management, and the stage of pregnancy of aborting females (with a higher ability to detect late abortions in dairy than beef cattle herds). Finally, based on mandatory abortion notification data, the mean lag between abortion occurrence and re-insemination is less than 105 days for 75% of aborted cows.

Despite these limitations, a syndromic surveillance system based on our indicator can contribute to improving bovine abortion surveillance. Theoretically, the current event-driven surveillance system covers the entire bovine population; however, it lacks sensitivity [10]. An evaluation of the ability of such a system to detect brucellosis in Japan (where the disease is believed to be eradicated) predicted that abortion surveillance would identify a first brucellosis outbreak within a median delay of 19 to 33 months depending on the reporting rate [50]. Despite some improvements, the effectiveness of the current mandatory abortion surveillance system will always depend on farmers’ and veterinarians’ willingness to participate and consequently under-reporting will remain its major limitation [51]. Moreover, event-driven surveillance is currently brucellosis-oriented, while the MAIR is a non-specific indicator that can detect anomalies associated with a wide range of abortive diseases [43].

With the objective of implementing a prospective syndromic surveillance system based on the MAIR, the next step requires careful investigation of the performances of the algorithm under alternative scenarios of disease patterns in order to maximize the sensitivity of the system and minimize the number of false alerts [42]. Further studies are also needed to extend this approach to late abortions.

According to the CDC, the timely monitoring of non-specific health indicators, so-called syndromic surveillance, is one of the ways to improve early detection of health events [52]. In animal health, AI data are collected in many countries according to cattle performance recording programs [53,54]. Our study indicates that they can be used to implement syndromic surveillance for mid-term abortions in cattle, with no additional workload for data providers. Such a system would complement the existing event-driven surveillance system by detecting changes or events that would otherwise not have been detected by traditional surveillance.
